# Comparison of rTMS and dTMS in TRD: evidence gaps, cost-effectiveness, and ethical priorities

**DOI:** 10.3389/fpsyt.2025.1722486

**Published:** 2026-01-07

**Authors:** Walter Paganin

**Affiliations:** Studio Psicologia Signorini, Guidonia, Lazio, Italy

**Keywords:** clinical decision-making, cost-effectiveness, depression, dTMS, evidence gap, neuromodulation, rTMS

## Introduction: a scientific and clinical paradox

Transcranial magnetic stimulation (TMS) represents one of the most significant innovations in treatment-resistant depression management over recent decades (1). Among available techniques, repetitive transcranial magnetic stimulation (rTMS) and deep transcranial magnetic stimulation (dTMS) constitute the most established options, both supported by efficacy and safety data. A recent systematic review highlights a substantial discrepancy between clinical adoption and the availability of direct comparative data (2). Despite the growing use of both technologies, only three studies have directly evaluated their relative efficacy, indicating a limited comparative evidence base. This lack of head-to-head data represents not only a scientific limitation but also a set of clinically relevant regulatory, ethical, and health-economic considerations. rTMS employs a figure-8 coil with more focal and relatively superficial stimulation (3), while dTMS utilizes an H-coil generating broader and deeper field penetration (4). Both techniques have achieved regulatory approval and have become increasingly integrated into routine clinical services. The central question has therefore shifted from establishing whether these interventions are effective, both have demonstrated superiority over sham, to determining which modality may be preferable for specific patient profiles and clinical scenarios.

## Regulatory framework: United States and Europe

In the United States, the Food and Drug Administration (FDA) distinguishes different approval pathways: Premarket Notification [510(k)]/*de novo* for low-to-moderate risk devices (based on substantial equivalence) and Premarket Approval (PMA) for Class III devices (high risk), requiring direct demonstration of safety and efficacy (5). rTMS obtained *de novo*/510(k) clearance in 2008 (NeuroStar, K083538) (6), while dTMS received 510(k) clearance in 2013 (BrainsWay H-coil, K122288), based on substantial technological equivalence (7). This pathway does not imply reduced effectiveness but indicates that its regulatory approval was based on device comparability rather than large-scale, modality-specific clinical trials (8).

In Europe, TMS devices obtain Conformité Européenne (CE) certification through risk-based classification: rTMS typically achieves Class IIa status (medium-low risk) reflecting decades of clinical experience with superficial stimulation, while dTMS may receive Class IIb classification (medium-high risk) due to greater energetic power and deeper brain penetration (9). Unlike FDA approval, European CE marking prioritizes technical and safety compliance over large-scale efficacy trials (10), facilitating faster market access but potentially establishing different evidentiary expectations for clinical validation. This regulatory divergence creates a paradox: both techniques achieve similar regulatory status despite fundamentally different approval pathways and evidence requirements, contributing to the perception of functional equivalence, which may overlook meaningful technical and effectiveness-related distinctions.

## Discussion: clinical evidence gap

It is notable that the entire comparative evidence base between rTMS and dTMS consists of only three methodologically limited studies (11–13). Critical limitations include geographic concentration (two Croatian studies) and restricted generalizability. Systematic reviews and the few existing head-to-head comparisons suggest similar ranges of efficacy and tolerability outcomes for rTMS and dTMS (2). Paradoxically the limited volume and heterogeneity of available data make it difficult to derive definitive conclusions regarding their relative clinical value. While both modalities have demonstrated effectiveness compared with sham, the absence of comparative cost-effectiveness assessments means that their relative economic efficiency remains undetermined (14). The broadly similar regulatory status of rTMS and dTMS in the United States, both FDA-cleared for major depressive disorder may contribute to the assumption of functional equivalence, potentially overlooking relevant differences in coil architecture, stimulation depth, and operational requirements. To contextualize the statement that only three comparative studies exist, **Table 1** summarizes their methodological characteristics, stimulation protocols, primary outcomes, and limitations. These studies represent the entirety of the direct evidence base available to date and highlight the need for adequately powered, multicenter, comparative trials.

**Table 1:** Comparative studies of rTMS vs dTMS in treatment-resistant depression and key protocol characteristics.

Study (Author, year)	Design & sample	rTMS protocol (Figure-8 coil)	dTMS protocol (H1-coil)	Primary outcomes	Key results	Methodological limitations
Wirecki et al., 2015	Observational, real-world; N = 66 (rTMS n=45; dTMS n=21). Single center.	Figure-8 coil; 10 Hz; 3000 pulses/session; 20 sessions over 4 weeks.	H1-coil; 18 Hz; 1980 pulses/session; 20 sessions over 4 weeks.	Beck Depression Inventory (BDI).	Both groups showed symptom reduction with no significant differences. Good tolerability in both arms.	Small, unbalanced sample; no randomization; no blinding; no long-term follow-up; single center.
Filipčić et al., 2018	Randomized, single-blind RCT; N = 76; 3 arms: H1-coil, figure-8 coil, standard therapy.	Figure-8 coil; 10 Hz; 3000 pulses; L-DLPFC target; 5 sessions/week × 4 weeks.	H1-coil; 18 Hz; 1980 pulses; broader/deeper field; 5 sessions/week × 4 weeks.	Short-term efficacy and tolerability; BDNF, MADRS, PSQI.	Both modalities reduced depressive symptoms; similar safety; exploratory biomarker signals not conclusive.	Small sample; single center; short follow-up; partial blinding; heterogeneous inclusion criteria; subjective secondary outcomes.
Filipčić et al., 2019	Randomized, single-blind controlled trial; N = 228; 3 arms: H1-coil, figure-8 coil, control.	Figure-8 coil; 10 Hz; 3000 pulses; standard TRD protocol; 20 sessions.	H1-coil; 18 Hz; 1980 pulses; deeper stimulation; 20 sessions.	Remission (HAM-D17 ≤7); response (≥50% reduction).	Remission: H1 60%, figure-8 43%; response rates significantly higher with H1; both active arms > control.	No sham control; different monitoring intensity vs control; single center; no long-term outcomes; non-standardized session parameters between modalities
Key technical distinctions (across all studies)		Focal stimulation; ~1–1.5 cm depth; higher positioning sensitivity; longer sessions; lower field spread	Broader and deeper field (~3–4 cm); lower positioning sensitivity; fewer pulses; higher energetic load; wider cortical engagement			Derived from protocol descriptions) and coil modeling

The technical characteristics of rTMS and dTMS differ in several fundamental ways that are relevant for interpreting the limited comparative evidence available. While rTMS typically employs focal stimulation delivered through figure-8 coils, dTMS uses H-coil systems designed to achieve deeper and broader field penetration. These differences imply distinct patterns of cortical engagement and may influence treatment accessibility, operational complexity, and clinical variability. A more detailed examination of protocol heterogeneity in rTMS and the specific technical limitations associated with the H-coil used in dTMS is provided in the following subsection.

## Technical limitations of dTMS and heterogeneity of rTMS protocols

The interpretation of comparative findings between rTMS and dTMS is complicated by the substantial heterogeneity of rTMS protocols currently in use. Contemporary clinical practice includes high-frequency L-DLPFC stimulation (10–20 Hz), low-frequency R-DLPFC stimulation (1 Hz), sequential bilateral stimulation, and multiple theta-burst variants such as intermittent TBS (iTBS) and continuous TBS (cTBS). These protocols differ in frequency, train duration, inter-train intervals, total pulse number, coil-positioning methods, stimulation intensity relative to motor threshold, and overall treatment duration (15, 16). This methodological variability contributes to differences in cortical engagement and introduces non-trivial variability in clinical outcomes, thereby limiting the interpretability of the few head-to-head comparisons with dTMS. In contrast, dTMS protocols are more standardized but exhibit specific technical limitations. The H1-coil generates deeper and broader magnetic fields, but at the cost of reduced focality, increased field dispersion, and limited ability to selectively stimulate defined cortical subregions. Its complex geometric structure and variable depth-of-stimulation may also introduce inter-individual variability in effective cortical penetration (17). These biophysical characteristics demonstrate that rTMS and dTMS deliver fundamentally different forms of neuromodulation, reinforcing the need to interpret comparative evidence in light of their distinct technical profiles.

## The economic void: clinical decisions without evidence

Despite the widespread adoption of both rTMS and dTMS, no comparative economic analyses exist to guide decisions regarding resource allocation, procurement, or reimbursement. The available literature provides only indirect economic evidence, primarily related to rTMS, leaving a substantial gap that limits a comprehensive evaluation of the relative value of these neuromodulation modalities. This gap is examined in detail in the following section.

## Integrated economic and health-policy considerations

Although rTMS and dTMS have become increasingly available in mental health services, no direct cost-effectiveness comparisons between the two modalities have been published to date, and the existing economic literature remains limited to indirect evaluations. Published health-technology assessments indicate that rTMS is generally associated with lower acquisition costs, lower maintenance requirements, and shorter operator training compared with dTMS, features that contribute to greater scalability in routine psychiatric practice (18). By contrast, dTMS relies on higher-energy devices and proprietary H-coil systems, resulting in substantially higher upfront and operational costs (2). Indirect economic studies suggest that rTMS may be cost-saving or cost-effective relative to pharmacotherapy alone, largely due to reductions in hospitalization rates, functional impairment, and long-term disability. No published evidence currently evaluates dTMS within a comparable economic framework, and no study has assessed its cost-effectiveness, cost-utility, or budget impact. Likewise, no analyses have compared rTMS and dTMS in terms of treatment duration, resource utilization, or drop-out rates, leaving these parameters uncharacterized for dTMS. From a health-policy perspective, the absence of comparative economic data means that reimbursement decisions, device procurement, and treatment availability are often driven by logistical constraints rather than by evidence-based evaluation. This dynamic may exacerbate geographic variability in access to neuromodulation, particularly in publicly funded systems where procurement must be justified in relation to demonstrated clinical and economic value. Without systematic cost-effectiveness analyses, including those specifically addressing dTMS service planning cannot determine whether its higher operational costs translate into measurable clinical or economic advantages over rTMS (2). This uncertainty generates an ethical paradox: healthcare systems may invest in more expensive technologies without proven additional benefit. A natural extension of these economic considerations concerns their ethical implications, as decisions made in the absence of robust comparative and cost-effectiveness data inevitably affect fairness, resource allocation, and the responsible use of healthcare resources.

## Ethical implications

Continuing clinical practice in the absence of adequate comparative evidence raises fundamental ethical issues. Is it justifiable to deploy costly technologies without clear evidence of differential benefit? This pertains to the principle of non-maleficence in the allocation of finite healthcare resources when relative therapeutic advantage remains insufficiently defined. Differences in access to dTMS, given its higher operational costs, are ethically relevant only if a consistent and clinically meaningful differential effect can be demonstrated, which current evidence has not yet established. Two studies may clarify whether dTMS offers clinical advantages:

- HAUVERDEEP (NCT04956016, France; n=152; scheduled completion in 2025): head-to-head comparison of conventional rTMS and dTMS, with efficacy endpoints assessed through day 90.
- ReDeeMD (NCT05902312, Canada; n=50; scheduled completion in 2027): head-to-head rTMS vs dTMS in treatment-resistant depression (TRD), including predictive biomarker analyses

However, while these studies may clarify potential clinical differences, they are unlikely to resolve the outstanding cost-effectiveness questions.

## Urgent priorities

The absence of pharmacoeconomic studies represents the most critical gap in the comparative landscape between rTMS and dTMS. No study has included formal cost-effectiveness evaluations, forcing clinical decisions and resource allocation to be based on logistical constraints, equipment availability, treatment duration, center resources, rather than on comparative economic data. The scientific community must address this evidence gap urgently. Immediate priorities include: multicenter randomized trials with adequate statistical power and long-term follow-up to evaluate duration of effects and relapse rates. Integrated pharmacoeconomic analyses must consider not only acquisition costs, but also maintenance, cycle duration, and impact on quality of life.

## Neuroinflammatory biomarkers

The use of predictive biomarkers may facilitate more personalized neuromodulation strategies in TRD. Inflammatory markers such as high-sensitivity C-reactive protein (hs-CRP ≥3 mg/L), IL-6, TNF-α, IL-1β, and related cytokines have been proposed to delineate an “inflamed TRD subtype” (19). However, despite their increasing application in patient stratification, these biomarkers must be interpreted with caution, as the relationship between peripheral inflammatory signatures and central neuroimmune mechanisms remains a matter of debate (20). This stratification may nonetheless be relevant for comparisons between rTMS and dTMS, given that deeper stimulation could theoretically modulate neuroinflammatory circuits more effectively than superficial approaches. Although such observations are promising, the available data remain exploratory and insufficient to inform differential selection between the two modalities. More rigorous studies integrating inflammatory biomarkers with clinical, neurophysiological, and connectivity measures are required to determine whether biological profiles can meaningfully guide treatment choice, including the possibility that individuals with inflammatory features may benefit preferentially from deeper neuromodulatory interventions (21).

## Functional connectivity

Resting-state functional connectivity between the left dorsolateral prefrontal cortex (DLPFC) and the subgenual anterior cingulate cortex (sgACC), measured by fMRI, represents the most robust predictive biomarker: patients with hypoconnectivity show response rates >70% to high-frequency DLPFC stimulation, while normal/hyperconnectivity responds less favorably (21, 22). Anatomical specificity assumes differential relevance: rTMS acts primarily on superficial prefrontal cortex, while dTMS with H1-coil reaches deeper regions of the fronto-limbic circuit, including the anterior cingulate cortex. Patients with extensive fronto-limbic network dysfunction could theoretically benefit from deep stimulation, a hypothesis requiring direct experimental validation (23).

## Multimodal integration and predictive algorithms

The optimal approach integrates multiple biomarkers into validated algorithms. The combination of hs-CRP, DLPFC-sgACC connectivity, and demographic-clinical parameters (age, episode duration, failed medications) stratifies patients into subgroups with differential response probabilities (24). Machine learning models on multimodal neuroimaging demonstrate predictive accuracy >80% in identifying TMS responders, surpassing traditional clinical prediction (25). Extension to direct rTMS/dTMS comparison represents an immediate research priority.

## Conclusions: the evidence imperative

The current approach to treatment-resistant depression requires a paradigm shift toward more comprehensive and individualized approaches. Neuromodulation techniques are a key pillar of this integrated approach. Transcranial Magnetic Stimulation (rTMS/dTMS) has demonstrated efficacy in patients unresponsive to pharmacotherapy. However, as highlighted in recent literature, a notable discrepancy remains: despite their broad integration into clinical practice, only three studies directly comparing rTMS and dTMS exist, creating a limited evidence base that constrains fully informed clinical decision-making and rational resource planning. This scarcity of comparative data represents not only a scientific limitation but also a set of ethical and health-economic considerations relevant to service delivery and equitable access. Integrating predictive biomarkers and functional connectivity patterns between the DLPFC and sgACC, may offer promising avenues for tailoring neuromodulation modalities to individual clinical profiles, thus enhancing the precision of treatment selection. Ongoing studies such as HAUVERDEEP and ReDeeMD may clarify differential clinical benefits, but the need for formal pharmacoeconomic evaluations remains urgent. This evolution in the treatment of treatment-resistant and difficult-to-treat depression will require not only the implementation of collaborative care models and systematic integration of psychosocial interventions, but also a rigorous commitment to generating comparative evidence for neuromodulation technologies.

This transformation represents both a clinical and an ethical priority for improving care in TRD. The journey requires sustained commitment from clinicians, researchers, healthcare systems, and policymakers. Only through such comprehensive efforts can we hope to address the growing challenge of treatment-resistant depression and provide hope to those who have found little benefit from current approaches.

Take-home message: A decade of clinical use supported by only three comparative studies underscores the need for a more robust and systematic evidence base.

## References

[1] Li H Cui L Li J Liu Y Chen Y . Comparative efficacy and acceptability of neuromodulation procedures in the treatment of treatment-resistant depression: a network meta-analysis of randomized controlled trials. J Affect Disord. (2021) 287:115–24. doi: 10.1016/j.jad.2021.03.019, PMID: 33780827

[2] Paganin W Contini LM . Comparison of therapeutic efficacy in depression between repetitive TMS and deep TMS. J Neural Transm. (2025) 132:1113–24. doi: 10.1007/s00702-025-02944-w, PMID: 40423727 PMC12479554

[3] Lefaucheur JP Aleman A Baeken C Benninger DH Brunelin J Di Lazzaro V . Evidence-based guidelines on the therapeutic use of repetitive transcranial magnetic stimulation (rTMS): An update (2014–2018). Clin Neurophysiol. (2020) 131:474–528. doi: 10.1016/j.clinph.2019.11.002, PMID: 31901449

[4] Rapinesi C Bersani FS Kotzalidis GD Imperatori C Del Casale A Di Pietro S . Maintenance deep transcranial magnetic stimulation sessions are associated with reduced depressive relapses in patients with unipolar or bipolar depression. Front Neurol. (2015) 6:16/abstract. doi: 10.3389/fneur.2015.00016/abstract 25709596 PMC4321576

[5] Health C for D and R. FDA. FDA . Premarket approval (PMA) (2023). Available online at: https://www.fda.gov/medical-devices/premarket-submissions-selecting-and-preparing-correct-submission/premarket-approval-pma. (Accessed October 8, 2025).

[6] Cheng CM Li CT Tsai SJ . Current updates on newer forms of transcranial magnetic stimulation in major depression. In: Kim YK , editor. Major depressive disorder, vol. 1305 . Springer Singapore, Singapore (2021). p. 333–49. doi: 10.1007/978-981-33-6044-0_18, PMID: 33834408

[7] Levkovitz Y Isserles M Padberg F Lisanby SH Bystritsky A Xia G . Efficacy and safety of deep transcranial magnetic stimulation for major depression: a prospective multicenter randomized controlled trial. World Psychiatry. (2015) 14:64–73. doi: 10.1002/wps.20199, PMID: 25655160 PMC4329899

[8] Health C for D and R. FDA. FDA . 510(k) clearances (2024). Available online at: https://www.fda.gov/medical-devices/device-approvals-and-clearances/510k-clearances. (Accessed October 8, 2025).

[9] Roth Y Amir A Levkovitz Y Zangen A . Three-dimensional distribution of the electric field induced in the brain by transcranial magnetic stimulation using figure-8 and deep H-coils. J Clin Neurophysiol. (2007) 24:31–8. doi: 10.1097/WNP.0b013e31802fa393, PMID: 17277575

[10] Jurczak KM van der Boon TAB Devia-Rodriguez R Schuurmann RCL Sjollema J van Huizen L . Recent regulatory developments in EU Medical Device Regulation and their impact on biomaterials translation. Bioeng Transl Med. (2024) 10:e10721. doi: 10.1002/btm2.10721, PMID: 40060767 PMC11883120

[11] Wirecki T Stephenson C Seale R Davis L . A Comparison of rTMS and dTMS Treatment Outcomes for Major Depressive Disorder in a Real-World Clinical Practice Setting. Brain Stimulat.. (2015) 8:e6. doi: 10.1016/j.brs.2015.07.022

[12] Filipcic I Milovac Z Sucic S Gajsak T Filipcic IS Ivezic E . Efficacy, safety and tolerability of augmentative rtms in treatment of major depressive disorder (mdd): A prospective cohort study in Croatia. Psychiatr Danub.. (2017) 29:31–8. doi: 10.24869/psyd.2017.31, PMID: 28291972

[13] Filipčić I Filipčić IŠ Gajšak T Milovac Ž Sučić S Ivezić E . Efficacy and safety of repetitive transcranial magnetic stimulation using an H1-coil or figure-8-coil in the treatment of unipolar major depressive disorder: A study protocol for a randomized controlled trial. Psychiatr Danub. (2018) 30:41–6. doi: 10.24869/psyd.2018.41, PMID: 29546857

[14] Mårtensson B Nordenskjöld A Johansson S Pettersson A Fundell S Moberg K . Behandling av depression med transkraniell magnetstimulering med H-spole (dTMS). Stockholm, Sweden: SBU (2020).

[15] Cotovio G Ventura F Rodrigues da Silva D Pereira P Oliveira-Maia AJ . Regulatory clearance and approval of therapeutic protocols of transcranial magnetic stimulation for psychiatric disorders. Brain Sci. (2023) 13:1029. doi: 10.3390/brainsci13071029, PMID: 37508962 PMC10377201

[16] Blumberger DM Vila-Rodriguez F Thorpe KE Feffer K Noda Y Giacobbe P . Effectiveness of theta burst versus high-frequency repetitive transcranial magnetic stimulation in patients with depression (THREE-D): a randomised non-inferiority trial. Lancet Lond Engl. (2018) 391:1683–92. doi: 10.1016/S0140-6736(18)30295-2, PMID: 29726344

[17] Worbs T Rumi B Madsen KH Thielscher A . Realistic electric field characterization of clinically used deformable large TMS coils in a large cohort. Brain Stimulat. (2025) 18:1174–83. doi: 10.1016/j.brs.2025.05.136, PMID: 40482811 PMC12419802

[18] Voigt J Carpenter L Leuchter A . Cost effectiveness analysis comparing repetitive transcranial magnetic stimulation to antidepressant medications after a first treatment failure for major depressive disorder in newly diagnosed patients - A lifetime analysis. PloS One. (2017) 12:e0186950. doi: 10.1371/journal.pone.0186950, PMID: 29073256 PMC5658110

[19] Osimo EF Baxter LJ Lewis G Jones PB Khandaker GM . Prevalence of low-grade inflammation in depression: a systematic review and meta-analysis of CRP levels. Psychol Med. (2019) 49:1958–70. doi: 10.1017/S0033291719001454, PMID: 31258105 PMC6712955

[20] Paganin W Signorini S . Inflammatory biomarkers in depression: scoping review. BJPsych Open. (2024) 10:e165. doi: 10.1192/bjo.2024.787, PMID: 39343996 PMC11536280

[21] Rajkumar RP . Immune-inflammatory markers of response to repetitive transcranial magnetic stimulation in depression: A scoping review. Asian J Psychiatry. (2024) 91:103852. doi: 10.1016/j.ajp.2023.103852, PMID: 38070319

[22] Fox MD Buckner RL White MP Greicius MD Pascual-Leone A . Efficacy of transcranial magnetic stimulation targets for depression is related to intrinsic functional connectivity with the subgenual cingulate. Biol Psychiatry. (2012) 72:595–603. doi: 10.1016/j.biopsych.2012.04.028, PMID: 22658708 PMC4120275

[23] Cash RFH Zalesky A . Personalized and circuit-based transcranial magnetic stimulation: evidence, controversies, and opportunities. Biol Psychiatry. (2024) 95:510–22. doi: 10.1016/j.biopsych.2023.11.013, PMID: 38040047

[24] Cearns M Hahn T Baune BT . Recommendations and future directions for supervised machine learning in psychiatry. Transl Psychiatry. (2019) 9:271. doi: 10.1038/s41398-019-0607-2, PMID: 31641106 PMC6805872

[25] Drysdale AT Grosenick L Downar J Dunlop K Mansouri F Meng Y . Resting-state connectivity biomarkers define neurophysiological subtypes of depression. Nat Med. (2017) 23:28–38. doi: 10.1038/nm.4246, PMID: 27918562 PMC5624035

